# Predictors of Peritoneal Surface Recurrence and Quantitative Association with Time to Relapse After Complete CRS/HIPEC for Colorectal Peritoneal Metastasis

**DOI:** 10.3390/cancers18020299

**Published:** 2026-01-19

**Authors:** Corey A. Hounschell, Aubrey C. Swilling, Sahaam Mirza, Katelyn Sanner-Dixon, Jill Haley, Luke V. Selby, Shahid Umar, Mazin Al-Kasspooles

**Affiliations:** 1 Providence Saint John’s Cancer Institute, Santa Monica, CA 90404, USA; corey.hounschell@providence.org; 2 Department of Surgery, University of Kansas Medical Center, Kansas City, KS 66211, USA; aswilling@kumc.edu (A.C.S.); smirza@kumc.edu (S.M.); ksannerdixon@kumc.edu (K.S.-D.); jhaley@kumc.edu (J.H.); lselby@kumc.edu (L.V.S.); sumar@kumc.edu (S.U.)

**Keywords:** peritoneal surface malignancy, CRS/HIPEC, colon cancer

## Abstract

Background/Objectives: Peritoneal surface metastases (PSMs) from colorectal cancer have high rates of peritoneal recurrence after cytoreductive surgery (CRS) and hyperthermic intraperitoneal chemotherapy (HIPEC). Prior studies dichotomize peritoneal recurrence into “early” and “late,” limiting insight into how clinicopathologic factors influence recurrence timing. This study aimed to identify predictors of peritoneal recurrence and quantify their continuous association with time to recurrence following CRS/HIPEC. Methods: Patients undergoing CC-0 CRS/HIPEC for colorectal PSM from 2018 to 2024 were identified from a prospectively maintained database. The primary outcome was peritoneal surface recurrence. Variables included peritoneal cancer index (PCI), tumor location, histology, HIPEC regimen, and KRAS/BRAF/SMAD4 status. Factors with *p* < 0.10 on univariable analysis were entered into multivariable logistic regression (recurrence: yes/no) and linear regression (time to recurrence). Results: Among 133 patients, 64 (48.1%) developed peritoneal recurrence. Median time to recurrence was 41.4 weeks (IQR 24.9–74.0), and PCI was higher among those who recurred (median 11.0 vs. 5.0, *p* < 0.01). Neither tumor stage, histology, intraperitoneal chemotherapy agent, nor molecular alterations were associated with increased risk of peritoneal recurrence. When controlling for PCI, right- and sigmoid-colon primaries independently predicted peritoneal recurrence compared to all other locations without influence on recurrence timing (right: OR 7.18; sigmoid: OR 6.54; *p* < 0.01). Among patients who recurred, each one-point increase in PCI corresponded to a 2.43-week earlier relapse (*p* < 0.01). Conclusions: Nearly half of patients with colorectal PSM recurred despite complete CRS/HIPEC. Tumor location predicted peritoneal recurrence, while PCI independently shortened time to relapse. Modeling PCI as a continuous predictor refines postoperative risk stratification and may inform individualized surveillance strategies.

## Simple Summary

Patients with colon cancer that has spread to the peritoneal cavity often undergo extensive surgery to remove all visible disease prior to the circulation of heated intraperitoneal chemotherapy. Despite this aggressive treatment, many patients experience peritoneal recurrence of disease. The factors that predict peritoneal recurrence and how they affect timing of recurrence are still poorly understood. In this study, patients treated at our center were reviewed to identify which features of their disease were linked to peritoneal recurrence and whether certain factors influenced how soon recurrence occurred. We found that both the original tumor location and amount of disease in the abdomen were important in predicting the likelihood of peritoneal recurrence. Importantly, we found that larger amounts of disease were linked to shorter time to peritoneal recurrence. These findings may help better estimate a patient’s individual risk and tailor surveillance strategies.

## 1. Introduction

Peritoneal surface metastasis (PSM) occurs in approximately 8–15% of patients with colorectal cancer and is associated with a distinct pattern of disease spread, a high risk of locoregional recurrence, and historically poor survival outcomes [1,2,3,4,5]. Cytoreductive surgery (CRS) combined with hyperthermic intraperitoneal chemotherapy (HIPEC) has emerged as a treatment modality capable of achieving long-term survival in select patients, particularly when completed macroscopic cytoreduction is achieved [6,7,8,9,10,11,12,13]. Despite these advances, peritoneal recurrence remains the most common pattern of failure after CRS/HIPEC, with nearly half of patients experiencing relapse in contemporary studies [14,15,16]. Understanding which patients are at greatest risk for peritoneal recurrence, as well as its likely timing, is therefore central to postoperative counseling, surveillance, and patient selection.

Several clinicopathologic variables have been linked to peritoneal recurrence risk after CRS/HIPEC, including peritoneal cancer index (PCI), completeness of cytoreduction score, tumor grade, and molecular alterations such as KRAS or BRAF status [7,14,17,18,19,20,21,22,23,24,25]. PCI reflects the extent of peritoneal tumor burden and remains one of the strongest predictors of peritoneal recurrence across multiple studies [7,17,18,22]. The biologic rationale underlying this association is multifactorial: higher tumor burden may indicate more aggressive intraperitoneal dissemination, greater microscopic residual disease despite complete cytoreduction, and an underlying tumor biology conducive to peritoneal spread [7,18,25]. Additionally, primary tumor location has emerged as a clinically meaningful determinant of metastatic behavior, with right-sided colon cancers exhibiting distinct mutational landscapes, mucinous differentiation, and predilection for peritoneal dissemination compared with left-sided tumors [26,27,28].

Although numerous studies have examined predictors of “early” versus “late” peritoneal recurrence after CRS/HIPEC, nearly all rely on arbitrary dichotomization of recurrence timing, typically defining “early” recurrence as occurring within 6–12 months of CRS/HIPEC [14,16,29]. This approach, while convenient, discards the continuous nature of time to relapse and limits the ability to quantify how specific variables, such as PCI, influence recurrence kinetics. As a result, the field lacks quantitative data describing how increasing tumor burden or other clinicopathologic features shift the timing of peritoneal relapse on a continuous scale. Understanding these relationships may refine postoperative risk stratification, guide surveillance intensity, and improve counseling for patients undergoing CRS/HIPEC.

To address these gaps, we conducted a retrospective analysis of patients undergoing complete CRS/HIPEC for colorectal PSM at a single institution over a 14-year period. The objectives of this study are twofold: to identify clinicopathologic factors associated with peritoneal surface recurrence, and to determine how these factors influence the continuous timing of peritoneal relapse among patients who recur. By quantifying the relationship between clinicopathologic variables and timing of peritoneal recurrence, we aim to provide a more nuanced assessment of postoperative risk beyond traditional binary recurrence endpoints.

## 2. Methods

A single-institution, prospectively maintained database was queried for patients who underwent CRS/HIPEC for colorectal PSM between 2018 and 2024. Patients were included in the analysis if they achieved a Completion of Cytoreduction score of 0 (CC-0). Cytoreduction included complete greater and lesser omentectomies in all patients and bilateral salpingo-oophorectomy in all female patients who retained fallopian tubes and ovaries. Complete mobilization of the right hepatic lobe and entry into the lesser sac were performed in all patients to allow for complete inspection of all peritoneal surfaces. Peritonectomy was limited to only regions with visible or palpable disease. Visceral resections were performed on a case-by-case basis with enteric reconstruction taking place after administration of intraperitoneal chemotherapy. Once complete cytoreduction was achieved, intraperitoneal chemotherapy was administered in a closed system with continuous agitation of the patient’s abdomen during perfusion. For those receiving Mitomycin-C, an initial dose of 15 mg/m^2^ in 2–4 L of 1.5% dextrose dialysis solution was administered, followed by an additional dose of 5 mg/m^2^ administered 45 min into perfusion. The solution was circulated for a total of 90 min at 42 °C. For those receiving Melphalan, one 60 mg/m^2^ dose of Melphalan was administered in 2–4 L of 1.5% dextrose dialysis solution and circulated for 60 min at 42 °C. Notably, patients at our institution with low-volume hepatic or lung metastasis may be offered CRS/HIPEC if they demonstrate long-term stability or response to systemic therapy before surgery. Patients with liver metastatic disease had their liver metastasis treated with non-anatomic wedge resection or ablation at the time of CRS/HIPEC. Metastatic lung lesions were monitored closely without treatment. Adjuvant therapy was administered to patients at the discretion of their medical oncologist.

Patient demographic, tumor, and treatment variables were captured. Cancer-specific variables included peritoneal cancer index (PCI), primary tumor location, histology, intraperitoneal chemotherapy agent, and molecular profiling, including KRAS, BRAF, and SMAD4 status. Administration of adjuvant chemotherapy is captured throughout our dataset as a dichotomous variable indicating if adjuvant therapy was received by the patient within 6 months after CRS/HIPEC as well as the agent used and duration of therapy. Patients included in the database underwent standardized surveillance after CRS/HIPEC: a physical exam and laboratory evaluation including CEA every 3 months for 2 years, every 6 months for years 3–5, and annually thereafter. Chest, abdomen, and pelvis CT with intravenous contrast was performed every 6 months for 5 years and then annually thereafter. Shorter-interval CT imaging was obtained on patients who developed symptoms, developed elevation of CEA, or had indeterminate findings on prior, routine CT imaging necessitating shorter-interval follow-up.

The primary outcome was peritoneal surface recurrence as a binomial variable. Covariates that were significant or close to significance on univariable analysis (Chi square, *p* < 0.10) were included in multivariable logistic regression to predict peritoneal surface recurrence. In the subset of patients who did have peritoneal surface recurrence, the secondary outcome was time to peritoneal surface recurrence measured in weeks, and a linear regression model was created to predict time to recurrence. Significance was set at α = 0.05 and analysis was performed in R studio.

## 3. Results

In total, 133 patients underwent CRS-HIPEC for CRC with CC-0 cytoreduction between 2018 and 2024. The median age at surgery was 58 years and patients were predominantly white (118/133, 88.7%) and female (82/133, 61.7%) (Table S1). Median overall survival for our cohort of patients was 51 months (95% CI: 42–71 months). The most common primary location for CRC was the right colon (54/133, 40.6%), followed by the sigmoid (42/133, 31.6%), left (10/133, 7.5%), and transverse colon (9/133, 6.8%). In total, 25 of the 133 patients (18.8%) in this study had extraperitoneal metastases. Isolated liver metastasis was present in 21 patients (15.7%), isolated lung metastasis in 1 patient (0.7%), and both liver and lung metastasis in 3 patients (2.3%). Less than 5% of patients had rectal primary tumors (6/133, 4.5%) or multiple primary tumors (3/133, 2.3%). The location of the primary tumor was unknown in 6.8% (9/133) (Table S2). A small subset of patients had signet ring histology (12/133, 9.0%). More than half of the cohort had a pathologic T4 stage at diagnosis (74/133, 55.6%). The median PCI score at the time of HIPEC was 8.00 (range 0–31) and the mean was 9.91 (SD: 7.19). Most patients received Mitomycin-C for intraperitoneal chemotherapy (95/133, 71.4%) and the remaining patients received Melphalan as part of a randomized clinical trial taking place at the time of their treatment (38/133, 28.6%). Most patients underwent adjuvant systemic therapy (76/133, 57.1%) of various regimens and durations (Table S2).

As most patients who undergo CRS/HIPEC at our center are referred from elsewhere, practice patterns in testing for specific tumor genetic alterations at the time of initial diagnosis varied widely. Alterations in KRAS were tested in 76.7% (102/133) of patients and of those tested, more than half (60/102, 58.8%) had variants detected. BRAF status was tested in fewer patients (93/103, 69.9%), and variants were detected in only 14.0% (13/93). SMAD4 was the least commonly assessed (37/133, 27.8%), and alterations were found in 9/37 patients (24.3%). Almost all patients had microsatellite stable disease (122/133, 91.7%) (Table S3).

Of the 133 patients, 64 patients (48.1%) had peritoneal surface recurrence. On univariate analysis, primary tumor stage, signet ring histology, HIPEC regimen, adjuvant systemic therapy, and molecular alterations in KRAS, BRAF, and SMAD4 were not associated with peritoneal surface recurrence or time to recurrence (Table 1). Patients who experienced peritoneal surface recurrence had significantly higher median PCI scores than those who did not recur (median [IQR] = 11.0 [7.0–16.0] vs. 5.0 [3.0–11.0], *p* < 0.01) (Table 1). The median time to peritoneal surface recurrence was 41.4 weeks (IQR: 24.9–74.0).

Patients with peritoneal surface recurrence were more likely to have a right-sided or sigmoid-colon primary tumor compared to those who did not experience recurrence (right-sided: 30/64, 46.9% vs. 24/69, 34.8%; sigmoid: 25/64, 39.1% vs. 17/69, 24.6%, *p* = 0.05) (Table 1). There was no difference in median time to peritoneal surface recurrence for patients with right-sided or sigmoid tumors (right-sided: 44.9 weeks vs. sigmoid: 39.7 weeks, *p* = 0.28).

**Figure 1:**
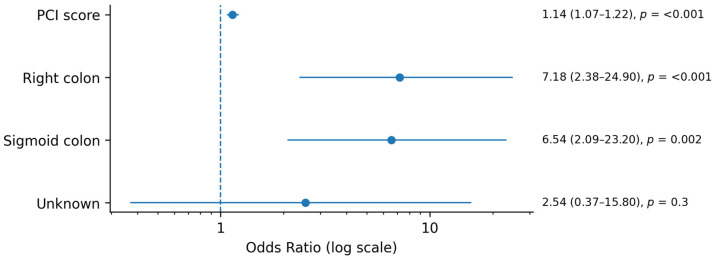
Forest plot of factors associated with recurrence risk.

**Figure 2:**
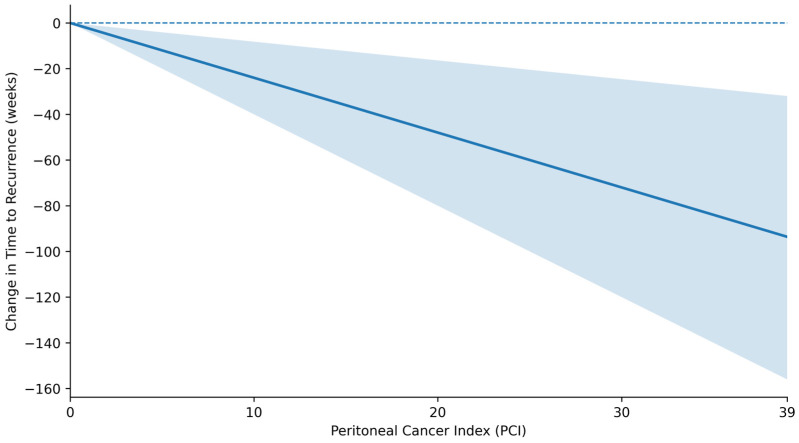
Continuous association between peritoneal cancer index (PCI) and time to recurrence.

In multivariate analysis, primary tumor location was categorized as right colon, sigmoid colon, or other. While an increase in PCI score was found to be an independent predictor for peritoneal surface recurrence in all tumor sites (OR 1.14, 95% CI 1.07–1.22, *p* < 0.01), controlling for PCI score revealed that a primary tumor location in the right or sigmoid colon was associated with increased odds of peritoneal surface recurrence compared to other primary colorectal sites (right: OR 7.18, 95% CI 2.38 −24.9, *p* < 0.01; sigmoid: OR 6.54, 95% CI: 2.09–23.2, *p* < 0.01) (Table 2, Figure 1). In the patients who did experience peritoneal surface recurrence, however, tumor location did not impact time to recurrence. An increase in PCI score of one point was found to be associated with earlier peritoneal recurrence of 2.4 weeks independent of primary tumor location (β = −2.43, *p* < 0.01) (Table 3, Figure 2). Sensitivity analysis was performed to exclude patients who recurred with PCI > 75th percentile. PCI remained a significant contributor to peritoneal recurrence, with each one-point increase in PCI being associated with 4.9-week earlier recurrence in this subset of patients (Table S4).

## 4. Discussion

In this single-institution cohort of patients who underwent complete CRS and HIPEC for colorectal PSM, we found that primary tumor location (right and sigmoid), as well as the extent of peritoneal disease at the time of surgery, quantified by PCI, was a strong determinant of risk of peritoneal recurrence. These results are consistent with earlier reports identifying PCI as a major prognostic factor after CRS/HIPEC for colorectal PSM [7,17,18,22,25], as well as those describing the prognostic implications of tumor location [26,30]. While these findings are relevant, the most important finding of this study is the association of increasing PCI with shorter time to relapse.

Prior studies have highlighted that a substantial proportion of PSM recurrences after CRS/HIPEC occur within the first one to two years after surgery [14,16,29], yet none have examined how tumor burden modulates the shape of the recurrence curve. The present study complements the findings of these prior studies by evaluating PCI as a continuous predictor of recurrence timing, rather than as a categorical or threshold-based variable. Our finding that higher PCI is not only associated with increased likelihood of relapse but also shorter time to relapse provides a more refined understanding of recurrence dynamics than traditional dichotomous “early” versus “late” endpoints. Because the primary objective was to quantify how PCI influences recurrence timing as a continuous process, we modeled time to recurrence using linear regression rather than Kaplan–Meier or Cox approaches. This strategy was intentionally chosen to avoid arbitrary categorization of recurrence timing (e.g., “early” vs. “late”) and to preserve the continuous nature of both PCI and time to relapse, allowing a more direct assessment of recurrence kinetics. This approach avoids the loss of information inherent to dichotomization and reflects an emerging methodological emphasis on modeling recurrence kinetics more precisely.

The clinical implications of these findings warrant careful interpretation. Although earlier recurrence was more common among patients with higher PCI, whether this should alter surveillance intensity or treatment strategy remains uncertain. Current surveillance approaches after CRS/HIPEC are largely center-dependent and not tailored to individualized risk profiles. While our data suggests that higher PCI may predict a shorter recurrence-free interval, these findings should be validated in larger, multi-institutional cohorts before informing risk-adapted surveillance protocols. Furthermore, this study must be interpreted in the context of its limitations. First, our sample size, while consistent with other single-institution series, limits the precision of subgroup analysis, particularly regarding the effects of primary tumor location. Molecular profiling was not available for all patients given many were referred from outside facilities with varying degrees of preoperative genetic testing, thus limiting the strength of our assessment of how specific mutations may influence recurrence timing. Previous studies have shown that RAS/RAF alterations adversely impact outcomes in CRS/HIPEC populations [19,20], but integrating comprehensive molecular data with continuous recurrence modeling remains an important future direction. Finally, while the majority of patients underwent surveillance at our institution with a defined and regimented surveillance strategy, some opted to undergo surveillance closer to their home, resulting in incomplete follow-up data in some cases. As a single-institution study, our findings are subject to institutional bias, particularly in pre- and postoperative treatment strategies, and may not be generalizable to other populations of patients within other regions. Importantly, however, the inclusion of select patients with limited extraperitoneal metastatic disease reflects real-world practice at our institution, where patients demonstrating durable stability or response to systemic therapy may be considered for CRS/HIPEC, and therefore may enhance the clinical applicability of these findings.

Future research should focus on validating our findings in large, prospective cohorts and integrating continuous recurrence modeling with molecular, radiographic, and histopathologic features. Identifying and describing multiple continuous predictors of recurrence and further refining recurrence prediction may support tailored surveillance strategies, improved patient counseling, and improved strategies for patient selection for CRS/HIPEC.

## 5. Conclusions

This study demonstrates that PCI is a key determinant of both recurrence risk and time to recurrence following CRS/HIPEC for colorectal PSM. By evaluating recurrence as a continuous process rather than relying on arbitrary dichotomization, our findings provide new insights into recurrence kinetics and highlight the interplay between disease burden and tumor biology. Further validation and integration of molecular and clinical variables will be essential to translating these findings into individualized postoperative management strategies.

## Figures and Tables

**Table 1:** Peritoneal surface recurrence after CRS-HIPEC.

	Yes	No	*p*-Value
**n**	64	69	
**Pathologic Tumor Stage (n (%))**			0.67
pT0	0 (0.0)	2 (2.9)	
pT2	3 (4.7)	2 (2.9)	
pT3	20 (31.2)	23 (33.3)	
pT4	37 (57.8)	37 (53.6)	
pTx	4 (6.2)	5 (7.2)	
**Signet Ring Histology (n (%))**			0.87
Yes	5 (7.8)	7 (10.1)	
No	59 (92.2)	62 (89.9)	
**KRAS Status (n = 102) (n (%))**			0.69
Detected	35 (61.4)	35 (61.4)	
Not detected/Wild type	22 (38.6)	22 (38.6)	
**BRAF Status (n = 93) (n (%))**			0.32
Detected	9 (18.4)	4 (9.1)	
Not detected/Wild type	40 (81.6)	40 (90.9)	
**SMAD4 Status (n = 37) (n (%))**			1.00
Detected	4 (23.5)	5 (25.0)	
Not detected/Wild type	13 (76.5)	15 (75.0)	
**HIPEC Regimen (n (%))**			0.40
Mitomycin-C	43 (67.2)	52 (75.4)	
Melphalan	21 (32.8)	17 (24.6)	
**Adjuvant Therapy (n(%))**	76 (57.1%)	57 (42.9)	0.92
**Primary Location (n (%))**			0.05
Right colon	30 (46.9)	24 (34.8)	
Transverse colon	3 (4.7)	7 (10.1)	
Left colon	2 (3.1)	8 (11.6)	
Sigmoid colon	25 (39.1)	17 (24.6)	
Rectum	1 (1.6)	5 (7.2)	
Multifocal	0 (0.0)	2 (2.9)	
Unknown	3 (4.7)	6 (8.7)	
**PCI Score (Median [IQR])**	11.00 [7.00, 16.00]	5.00 [3.00, 11.00]	<0.01

**Table 2:** Regression model: recurrence risk.

Characteristic	OR	95% CI	*p*-Value
PCI score	1.14	1.07–1.22	<0.001
Right colon	7.18	2.38–24.9	<0.001
Sigmoid colon	6.54	2.09–23.2	0.002
Unknown	2.54	0.37–15.8	0.3

**Table 3:** Time to recurrence (weeks).

Characteristic	Beta	95% CI	*p*-Value
PCI score	−2.4	−4.0 to −0.82	0.004
Right colon	−1.3	−41 to 38	>0.9
Sigmoid colon	−10	−50 to 30	0.6
Unknown	5.5	−57 to 68	0.9

## Data Availability

Data are contained within the article.

## Supplementary Materials

The following supporting information can be downloaded at https://www.mdpi.com/article/10.3390/cancers18020299/s1: Table S1. Patient Demographics. Table S2. Cancer Characteristics. Table S3. Molecular Profiling. Table S4. Time to Recurrence (PCI < 16).
