# Supplementary material for: Predictors of Peritoneal Surface Recurrence and Quantitative Association with Time to Relapse After Complete CRS/HIPEC for Colorectal Peritoneal Metastasis
Source: Cancers (Basel). 2026 Jan 19;18(2):299. doi: 10.3390/cancers18020299 (PMC12838633; doi:10.3390/cancers18020299)
Supplement: Supplementary file 1 [file cancers-18-00299-s001.zip › cancers-4060054-supplementary.pdf]

Supplementary material

Table S1. Patient Demographics.

Characteristic	Overall (N=133)
Age Mean (SD)	57.0 (11.4)
Age Median [Min, Max]	58.0 [18.0, 80.0]
Female	82 (61.7%)
Male	51 (38.3%)
Asian	5 (3.8%)
Black	5 (3.8%)
Other	5 (3.8%)
White	118 (88.7%)

Table S2. Cancer Characteristics.

Characteristic	Overall (N=133)
Left colon	10 (7.5%)
Multifocal	2 (1.5%)
Rectum	6 (4.5%)
Right colon	54 (40.6%)
Sigmoid colon	42 (31.6%)
Transverse colon	10 (7.5%)
Extraperitoneal metastasis	25 (18.8%)
Unknown	9 (6.8%)
pT0	2 (1.5%)
pT2	5 (3.8%)
pT3	43 (32.3%)
pT4	74 (55.6%)
pTx	9 (6.8%)
Signet Ring Histology Yes	12 (9.0%)
Mean PCI (SD)	9.91 (7.19)
Median PCI [Min, Max]	8.00 [0–31]
Melphalan	38 (28.6%)
Mitomycin-C	95 (71.4%)
Adjuvant systemic therapy (yes)	76 (57%)

Table S3. Molecular Profiling.

Characteristic	Overall (N=133)
KRAS Detected	60 (45.1%)
KRAS Wild/Not detected	42 (31.6%)
KRAS Missing	31 (23.3%)
BRAF Detected	13 (9.8%)
BRAF Wild/Not detected	80 (60.2%)
BRAF Missing	40 (30.1%)
SMAD4 Detected	9 (6.8%)
SMAD4 Wild/Not detected	28 (21.1%)
SMAD4 Missing	96 (72.2%)
MSI Stable	122 (91.7%)
MSI Unknown	3 (2.3%)
MSI Unstable	8 (6.0%)

Table S4. Time to Recurrence (PCI <16).

Characteristic	Beta	95% CI	p-value
PCI Score	-4.9	-8.6 to -1.2	0.013
Right colon	4.9	-57 to 66	0.9
Sigmoid colon	3.5	-60 to 67	>0.9
Unknown	4.9	-109 to 118	>0.9
